# Clinical characterisation and risk stratification of patients with arrhythmogenic right ventricular dysplasia/cardiomyopathy ≥50 years of age

**DOI:** 10.1007/s12471-016-0886-7

**Published:** 2016-08-31

**Authors:** M. J. van der Pols, T. P. Mast, P. Loh, J. F. van der Heijden, M. J. Cramer, R. N. Hauer, A. S. J. M. te Riele

**Affiliations:** 1 Department of Cardiology, University Medical Center Utrecht, Utrecht, The Netherlands; 2 Department of Cardiology, Johns Hopkins University School of Medicine, Baltimore, MD USA; 3 Netherlands Heart Institute, Utrecht, The Netherlands

**Keywords:** Arrhythmogenic right ventricular dysplasia, Cardiomyopathy, Elderly, Ventricular arrhythmia, Clinical phenotype

## Abstract

**Purpose:**

With the increased use of genetic testing for arrhythmogenic right ventricular dysplasia/cardiomyopathy (ARVD/C), this disease is being increasingly recognised among elderly patients. However, elderly ARVD/C patients were underrepresented in prior cohorts. We aimed to describe the phenotypical characteristics and outcomes among ARVD/C patients surviving ≥50 years.

**Methods:**

We assessed detailed phenotypical data of 29 patients who (1) presented at ≥50 years of age; and (2) fulfilled 2010 Task Force Criteria (TFC) for ARVD/C by last follow-up. Primary outcome was the occurrence of a major ventricular arrhythmia (sudden cardiac death, resuscitated sudden cardiac arrest or sustained ventricular tachycardia).

**Results:**

The majority (55 %) of elderly ARVD/C subjects were male, with a mean age of 59.0 ± 5.8 years at presentation. Study participants fulfilled a median of six (IQR 5–8) TFC criteria by last follow-up, of which arrhythmia criteria were most frequent (97 %), followed by structural criteria (83 %), depolarisation criteria (72 %) and repolarisation criteria (69 %)*. *By last follow-up, 15 (52 %) patients had experienced major ventricular arrhythmias. Most patients (*n* = 12) presented with this arrhythmia, while three experienced the event during 5.4 ± 3.2 years of follow-up. Compared with patients without an arrhythmic event, patients with major arrhythmias were more likely to be proband (*p* < 0.001) and male (*p* = 0.042). Likewise, survival free from sustained ventricular arrhythmia was lower among probands and males.

**Conclusion:**

Phenotypic characteristics of elderly ARVD/C patients are characterised by depolarisation abnormalities and structural cardiac changes. Ventricular arrhythmias in this elderly cohort are associated with male gender and proband status.

## Introduction

Arrhythmogenic right ventricular dysplasia/cardiomyopathy (ARVD/C) is an inherited cardiomyopathy characterised by fibrofatty replacement of predominantly the right ventricular myocardium, frequent ventricular arrhythmias, and an increased risk of sudden cardiac death [1, 2]. ARVD/C patients classically present between the second and fifth decade of life with symptomatic ventricular arrhythmias [3]. However, with increased use of molecular genetic testing for ARVD/C, the disease is more frequently recognised among elderly patients. Objective data describing the phenotypic characteristics and clinical course of elderly ARVD/C patients are currently lacking, which complicates recommendations for the management of this growing group of patients.

The objectives of this study were twofold. First, we aimed to describe the phenotypical characteristics and clinical course in a cohort of patients presenting with ARVD/C at the age of 50 years or older. Second, we sought to identify predictors of sustained ventricular arrhythmias that would be of value in determining which ARVD/C patients would benefit from more intensive screening or prophylactic implantation of an implantable cardioverter-defibrillator.

## Methods

### Study population

**Fig. 1:**
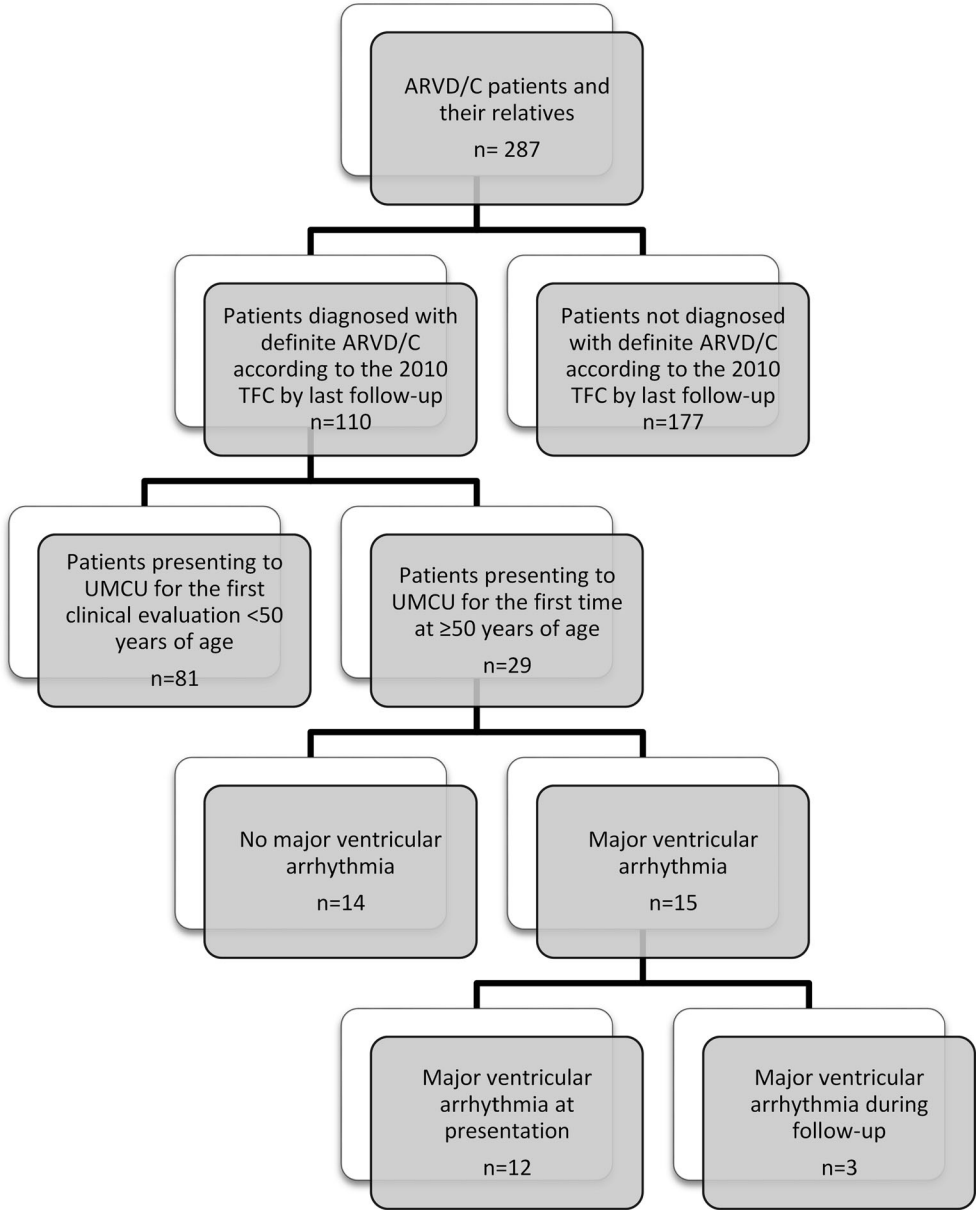
Ascertainment of the study population (*UMCU* University Medical Center Utrecht)

The study population was recruited from the University Medical Center Utrecht ARVD/C registry [4]. We identified 29 patients enrolled in the registry who (1) presented for first clinical evaluation at ≥50 years, and (2) were diagnosed with definite ARVD/C in accordance with the 2010 Task Force Criteria (TFC) by last follow-up [5]. Ascertainment of the study population is shown in Fig. 1.

### Clinical evaluation

Patients were evaluated as described previously [6]. Mutation analysis of the desmosomal genes encoding plakophilin-2 (*PKP2*), desmoplakin (*DSP*), desmoglein-2 (*DSG2*), desmocollin-2 (*DSC2*), and plakoglobin (*JUP*), and the non-desmosomal gene phospholamban (*PLN*) was performed in all patients, as reported previously [6, 7]. Criteria for pathogenicity of variants were determined as done previously [4]. Twelve-lead electrocardiograms (ECGs) (recorded at rest, 10 mm/mV at paper speed 25 mm/s) were evaluated for repolarisation (precordial T‑wave inversion in V1–2 or beyond) and/or depolarisation (epsilon waves or terminal activation duration ≥55 ms) criteria for ARVD/C. All patients were off drugs that influence ventricular depolarisation or repolarisation at the time of ECG recording. Twenty-four hour Holter monitoring was used to determine the frequency of premature ventricular complexes (PVC). Exercise stress testing was performed to detect ventricular tachycardia (VT) and ischaemia. Since signal-averaged ECG was not available in this cohort, we used invasive electrophysiological study as a measure of late potentials. Electrophysiological studies were performed in 15 subjects, the majority of whom (11/15, 73 %) had prior VT, while the remainder (4/15, 27 %) were symptomatic and had frequent ectopy on Holter monitoring. Late potentials on electrophysiological study were defined as local electrogram signals after termination of the QRS complex on the surface ECG during sinus rhythm. Echocardiography, cardiac magnetic resonance (CMR) imaging, and right ventricular angiography were reviewed to determine cardiac structural abnormalities. Per study design, all study participants fulfilled diagnostic criteria for definite ARVD/C according to the revised 2010 TFC [5].

### Outcome

The primary outcome measure was the occurrence of a major ventricular arrhythmia, which was a composite measure of the occurrence of sudden cardiac death (SCD), resuscitated sudden cardiac arrest (SCA), or spontaneous sustained VT (≥100/min, lasting ≥30 s or shorter with electrical or pharmacological interruption), as done previously [8]. In subjects with multiple endpoints, the first event was considered to be the censoring event.

### Statistical analysis

Continuous data are displayed as mean ± standard deviation or median (interquartile range [IQR]) and as proportions for categorical variables. Differences in continuous data were calculated using the independent samples *t*-test or Mann-Whitney *U* test as appropriate; for categorical data the Chi-square test or Fisher’s exact test was used. Kaplan-Meier curves were calculated for time to an event of a major ventricular arrhythmia. *P*-values <0.05 were considered significant. Data were analysed using SPSS software (version 22.0 for Mac).

## Results

### Study population

**Fig. 2:**
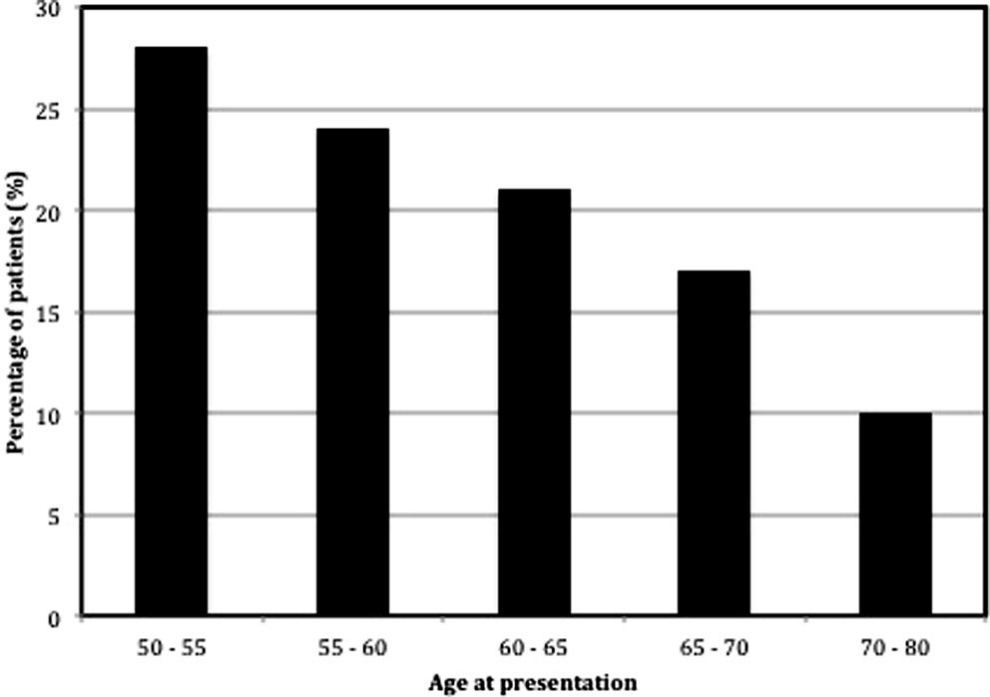
Age distribution of the study population. The distribution of age at first clinical cardiac evaluation among the study population. Overall, 28 % presented at 50–55 years, 24 % at 55–60 years, 21 % at 60–65 years, 17 % at 65–70 years and 10 % at 70–80 years of age. Mean age at time of presentation was 59.0 ± 5.8 (range 50.2–77.9) years

We included 29 patients who presented at ≥50 years of age and were diagnosed with ARVD/C by last follow-up. Their phenotypical characteristics are summarised in Table 1. Overall, 16 (55 %) study participants were male, with a mean age of 59.0 ± 5.8 years at the time of presentation. As shown in Fig. 2, the majority (*n* = 15, 52 %) of patients presented between the age of 50 and 60 years. ARVD/C-associated pathogenic mutations were found in 21 (72 %) patients; mutations were most frequently observed in the plakophilin-2 (*n* = 14, 48 %) gene, while the remainder harboured a phospholamban mutation (*n* = 7, 24 %).

**Table 1:** Phenotypic characteristics of the study population

	Definite ARVD/C (*n* = 29)
*Demographics*	
Male	16 (55)
Age at presentation (years)	59.0 ± 5.8
Proband	16 (55)
Mutation carrier	21 (72)
Plakophilin-2	14 (48)
Phospholamban	7 (24)
*Clinical phenotype at last follow-up*	
Age at diagnosis (years)	59.9 ± 6.7
Repolarisation TFC	20 (69)
T-wave inversion V1–3	16 (55)
T-wave inversion V1–2	2 (7)
T-wave inversion V4–6	2 (7)
T-wave inversion V1–4 with complete RBBB	0 (0)
Depolarisation TFC	21 (72)
Epsilon wave	5 (17)
>TAD	17 (59)
Late potentials	8/18 (44)
Arrhythmia TFC	28 (97)
Holter abnormal	16/21 (76)
PVC count; median [IQR]	2056 [560–5952]
LBBB superior axis VT	13 (45)
LBBB VT	15 (52)
Structural TFC	24 (83)
Major	21 (73)
Minor	3 (10)
TFC points; median [IQR]	6 [5–8]

*ARVD/C* Arrhythmogenic right ventricular dysplasia/cardiomyopathy, *RBBB* right bundle branch block, *IQR* interquartile range, *LBBB* left bundle branch block, *PVC* premature ventricular complex, >*TAD* prolonged terminal activation duration, *TFC* Task Force Criteria, *VT* ventricular tachycardia

### Clinical evaluation

The majority (*n* = 23, 79 %) of patients fulfilled the diagnostic TFC at the time of first presentation; the remainder (*n* = 6, 21 %) were diagnosed on average 3.6 ± 2.2 years after presentation. Reasons for clinical presentation were sustained monomorphic VT in 12 (41 %) patients, family screening in 10 (34 %) patients, and syncope/presyncope or palpitations in 7 (24 %) patients. No study subjects presented with an SCD or resuscitated SCA.

As shown in Table 1, T‑wave inversions were observed in 20 (69 %) elderly patients, most commonly (*n* = 16, 55 %) in leads V1–3. Depolarisation abnormalities were observed in 21 (72 %) patients, of which terminal activation duration ≥55 ms was the most frequent (*n* = 17, 59 %). Twenty-four hour Holter monitoring showed >500 PVCs in 16/21 subjects (76 %). The majority of patients (*n* = 24, 83 %) had structural abnormalities on imaging studies. The median TFC score was 6 (IQR 5–8) at last follow-up.

### Ventricular arrhythmia

Among the overall population, 15 (52 %) patients experienced a major ventricular arrhythmia, either at first presentation (*n* = 12, 80 %) or during follow-up (*n* = 3, 20 %). All first major ventricular arrhythmias presented with spontaneous sustained VT. Clinical characteristics of these patients are shown in Table 2. Compared with subjects without arrhythmia, patients who experienced an arrhythmic event were more likely to be male (*n* = 11 [73 %] vs. *n* = 5 [36 %]; *p* = 0.042) and proband (*n* = 13 [87 %] vs. *n* = 3 [21 %]; *p* < 0.001). There were no other differences in demographics or in any domain of the TFC between subjects with and without arrhythmia.

**Table 2:** Phenotypic characteristics of the study population

	VT (*n* = 15)	No VT (*n* = 14)	*p value*
*Demographics*			
Male	11(73)	5 (36)	0.042
Proband	13 (87)	3 (21)	<0.001
Mutation carrier	10 (67)	11 (79)	0.682
Plakophilin-2	7 (47)	7 (50)	0.862
Phospholamban	3 (20)	4 (29)	0.682
Mode of presentation			
Family screening Symptomatic Sustained VT SCA SCD	0 (0) 3 (20) 12 (80) 0 (0) 0 (0)	10 (71) 4 (29) 0 (0) 0 (0) 0 (0)	<0.001 0.682 <0.001 – –
*Clinical phenotype at last follow-up*			
Age at diagnosis (years)	62.5 ± 9.7	60.9 ± 6.4	0.623
T-wave inversion V1–3	7 (47)	9 (64)	0.340
T-wave inversion V1–2	2 (13)	0 (0)	0.483
T-wave inversion V4–6	0 (0)	2 (14)	0.224
T-wave inversion V1–4 with complete RBBB	0 (0)	0 (0)	–
Epsilon wave	4 (27)	1 (7)	0.330
>TAD	9 (60)	8 (57)	0.876
Late potentials	5/11 (45)	3/7 (43)	1.000
Holter monitor abnormal	5/8 (63)	11/13 (85)	0.325
PVC count; median [IQR]	919 [341–2095]	3108 [1205–6141]	0.205
Major structural TFC	13 (87)	8 (57)	0.109
Minor structural TFC	1 (7)	2 (14)	0.598
TFC points; median [IQR]	6[5–7]	6 [5–8]	0.506

*RBBB* right bundle branch block, *IQR* interquartile range, *PVC* premature ventricular complex, *SCA* sudden cardiac arrest, *SCD* sudden cardiac death*, >TAD* prolonged terminal activation duration, *TFC* Task Force Criteria, *VT* ventricular tachycardia

In elderly patients, an obvious arrhythmic substrate may occur in the setting of coronary artery disease (CAD). Electrocardiographic evidence of previous myocardial infarction was absent in all patients. In addition, we evaluated the presence or absence of CAD using coronary angiography and exercise stress testing in 15 patients with ventricular arrhythmia. Two (13 %) of these patients had evidence of CAD, defined as >50 % stenosis on coronary angiography. The first subject presented at age 77 with left bundle branch block (LBBB) inferior axis VT, and was found to have a 70 % stenosis in the circumflex artery on coronary angiography. CMR revealed right ventricular abnormalities suggestive of ARVD/C, and further evaluation revealed T‑wave inversions in V1-4 and a radical mutation in the plakophilin-2 gene (deletion exon 1–14). The second subject presented at 76 years of age with VT of LBBB superior axis morphology and a normal left ventricular ejection fraction of 54 %. However, a 70 % stenosis in margo obtusus 1 was found and subsequently treated with a bare metal stent. The LBBB superior axis VT recurred, after which another percutaneous coronary intervention was performed. After several years of recurrent VT, a comprehensive follow-up revealed ARVD/C based on late potentials, major structural CMR abnormalities, and LBBB superior axis VT. All other patients had no evidence of CAD on coronary angiography (*n* = 11) and/or exercise stress testing (*n* = 14).

### Ventricular arrhythmia during follow-up

**Fig. 3:**
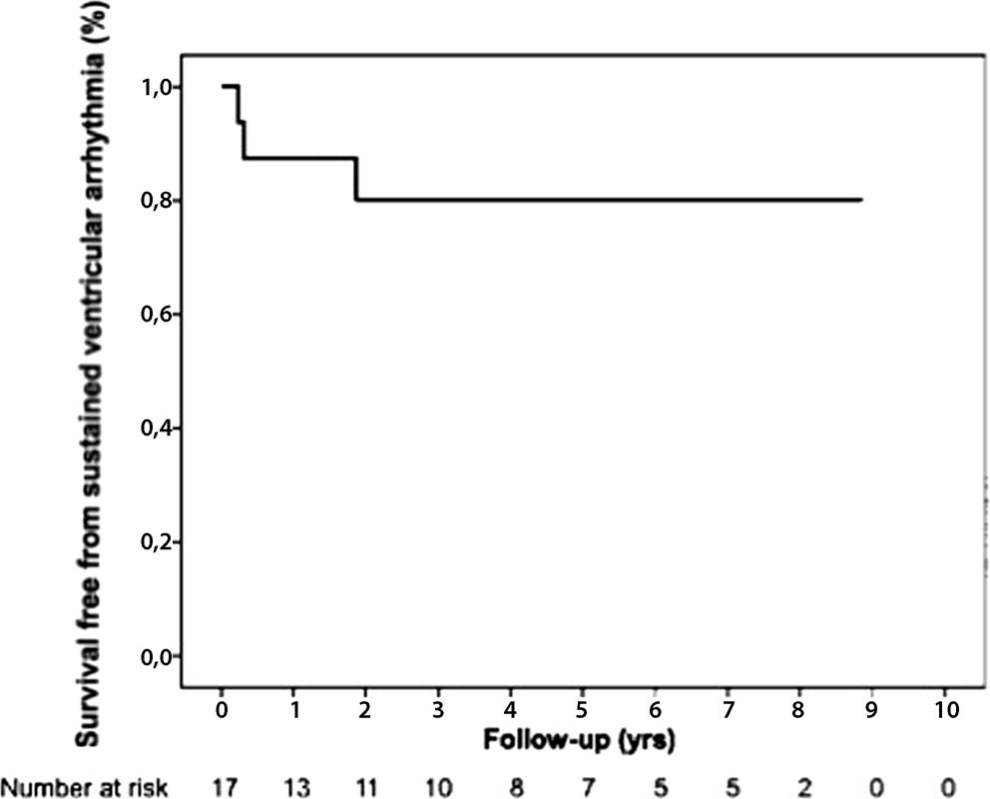
Survival free from major ventricular arrhythmias among the study population. Kaplan-Meier survival analyses demonstrating time to first major ventricular arrhythmia during follow-up among the overall cohort

Fig. 3 shows cumulative survival free from major ventricular arrhythmia in 17 patients who did not present with an arrhythmia. Over a mean follow-up of 5.4 ± 3.2 years, 3/17 (18 %) patients experienced a sustained monomorphic VT. Median cycle length of the VT was 240 ms (range 231–429). VT episodes showed LBBB morphology: two with superior, one with inferior axis. As shown in Fig. 3, median time to first VT was 4 months after first cardiological evaluation. Cumulative survival free from major ventricular arrhythmia after 6, 12 and 24 months was 88 % (95 % CI 72.3–100.0), 88 % (95 % CI 72.3–100.0), and 80 % (95 % CI 60.4–99.6), respectively.

The characteristics of the three patients who experienced an arrhythmic event during follow-up are shown in Table 3. The first event was experienced at a median age of 59 (range 55–69) years. Interestingly, all patients with arrhythmia were male and proband, and one of them was a carrier of an ARVD/C-associated pathogenic mutation (plakophilin-2). Prior to the arrhythmia, all three patients had reported symptoms including exercise-induced syncope (*n* = 2), palpitations (*n* = 2), and/or presyncope (*n* = 1). At the time of the arrhythmia, all patients fulfilled the TFC for ARVD/C: repolarisation criteria were fulfilled in 1 patient (major), depolarisation criteria in 2 patients (1 major, 1 minor), and arrhythmia criteria in 3 patients (2 major, 1 minor) (Table 3). In addition, cardiac imaging showed that all 3 subjects had major structural abnormalities. Distribution of age at diagnosis (*p* = 0.885), sex (*p* = 0.516) and presence of a pathogenic mutation (*p* = 0.242) was similar in patients with arrhythmic events at presentation and during follow-up.

**Table 3:** Characteristics of three patients experiencing first VT episode during follow-up

	Patient #1	Patient #2	Patient #3
*Demographics*			
Sex	M	M	M
Age at time of diagnosis (years)	55	69	59
Proband	Yes	Yes	Yes
Pathogenic mutation	–	–	+ (*PKP2*)
*Sustained arrhythmia characteristics*			
Age at time of arrhythmia	55	71	59
Morphology	LBBB	LBBB	LBBB
Axis	Superior	Superior	Inferior
Cycle length (ms)	240	231	429
Documentation	Walking	Cardiac stress test	Holter monitoring
*Clinical phenotype at time of sustained arrhythmia*			
Repolarisation TFC	None	None	TWI V_1_–_4_ (major)
Depolarisation TFC	None	Late potentials (minor)	Epsilon wave (major)
Arrhythmia TFC	LBBB superior axis VT (major)	560 PVCs/24 h (minor), LBBB superior axis VT (major)	11180 PVCs/24 h (minor), LBBB inferior axis VT (minor)
Structural TFC	RV aneurysm + reduced RV function (major)	RV aneurysm + reduced RV function (major)	RV aneurysm + reduced RV function (major)
Family history TFC	None	None	Major
TFC points at time of arrhythmic event	4	5	8
*Clinical features*			
Symptoms prior to event	Exercise-induced syncope, presyncope	Palpitations	Palpitations
Medication at time of arrhythmia	Perindopril 8 mg, Hydrochlorothiazide 25 mg	Atenolol 25 mg	Acenocoumarol^a^, Enalapril 20 mg, Simvastatin 40 mg
Relevant comorbidity	Hypertension	–	Ischaemic CVA
Coronary angiography	Normal	Normal	Normal

*CVA* cerebrovascular accident, *LBBB* left bundle branch block, *PKP2* Plakophilin-2, *PVC* premature ventricular complex, *TFC* Task Force Criteria, *TWI* T-wave inversion, *VT* ventricular tachycardia

^a^ Indication for acenocoumarol was a history of ischaemic CVA

## Discussion

This study has several interesting results. First, we found that the clinical phenotype of elderly ARVD/C patients is characterised by depolarisation abnormalities on 12-lead ECG, frequent PVCs on Holter monitoring, and structural alterations. Molecular genetic testing revealed a high percentage of phospholamban *p*.Arg14del mutation carriers. Second, risk of SCD was low in this elderly cohort, since none of the patients in this study experienced SCD/SCA. Last, arrhythmic events in this elderly cohort were associated with proband status and male gender, and all three patients experiencing events during follow-up were male and proband. These results provide interesting information for clinical practice, which is still largely based on expert opinion and consensus documents.

### Clinical phenotype in elderly patients

Prior studies have extensively described phenotypical characteristics in ARVD/C patients [3, 7, 9]. As such, we know that depolarisation and repolarisation changes on 12-lead ECG and frequent ectopy on Holter monitoring are often observed in ARVD/C patients [3, 7, 8]. However, it is important to note that elderly patients were underrepresented in prior ARVD/C cohorts, which typically had a mean age between 20–40 years [3, 7, 9]. To the best of our knowledge, the present study is the first to specifically focus on those ARVD/C patients who only come to clinical attention after the age of 50 years. As in younger ARVD/C subjects, ECG abnormalities, frequent ventricular ectopy, and structural alterations are commonly observed in this elderly cohort.

It is important to recognise that ventricular arrhythmias in the elderly population have a broad differential diagnosis and CAD, in particular, is much more prevalent than ARVD/C. Even in the 76-year-old patient in our ARVD/C cohort, monomorphic VT was initially thought to be related to the 70 % stenosis in a coronary artery. However, monomorphic VT in CAD in the absence of a previous myocardial infarction is very rare. Although our study was not designed to compare the clinical characteristics of elderly subjects with and without ARVD/C, these results provide a cautionary note to cardiologists, who may wrongly disregard the diagnosis of ARVD/C based on older age at presentation. On the other hand, wall motion abnormalities of the right ventricle are common and cardiologists should be aware that non-pathological right ventricular wall motion disorders can easily be mistaken for a pathological regional wall motion contraction, particularly in patients with ARVD/C [10]. Future studies should address specific treatment recommendations with regard to coronary artery disease and its prevention among ARVD/C patients.

### Genotype of elderly ARVD/C subjects

The yield of molecular genetic testing in ARVD/C typically lies between 50–63 % [7]. Our yield of 72 % is higher than in previously published reports. This may be due to a founder effect of the phospholamban *p*.Arg14del mutation [11], which was observed in a remarkably large subset of our population (24 %). Prior reports have shown that ARVD/C patients who harbour a phospholamban mutation present at an older age, frequently with left ventricular involvement [12, 13]. In this regard, it is interesting that only 62 % of our population had T‑wave inversion in the right precordial leads, in contrast to >85 % in the ARVD/C literature [3, 7, 8]. This may reflect a left predominant phenotype dominated by phospholamban mutations in our population.

### Elderly patients have a low risk of SCD

Prevention of SCD is the most important therapeutic goal in ARVD/C [14]. Therefore, it is an important finding of our study that none of the elderly patients in our cohort experienced SCD/SCA either at presentation or during follow-up. This is in contrast to prior studies with young ARVD/C patients which reported a 3–22 % rate of SCD [3, 4, 7]. This finding is particularly interesting in the context of recent basic science studies showing that desmosomal mutations lead to reduced sodium current which promotes re-entry susceptibility at an early disease stage [15–17]. In contrast, heterogeneous fibrofatty replacement of the myocardium leads to monomorphic sustained VT that is haemodynamically stable and can be adequately treated. In our study population, 82 % already had structural abnormalities on cardiac imaging. As such, we believe that our study subjects were more likely to experience haemodynamically well-tolerated monomorphic VT, rather than ventricular fibrillation. Future studies are necessary to determine whether a true difference in arrhythmic substrate exists in younger vs. elderly ARVD/C subjects.

### Clinical characteristics in subjects with VT

Despite a low risk of SCD, malignant ventricular arrhythmias are not uncommon among elderly ARVD/C patients. Our study shows that more than half of ARVD/C patients who present beyond 50 years of age experience a ventricular arrhythmia during more than five years of follow-up. Almost all of these events occurred at presentation. As such, the risk of ventricular arrhythmias seems highest for as yet unrecognised cases. Ventricular arrhythmias were significantly associated with male gender and proband status, and arrhythmic events during follow-up occurred exclusively among male probands. These data suggest that further screening and invasive risk stratification in elderly ARVD/C subjects may be most beneficial among male probands.

### Study limitations

This study was limited by its small sample size. However, this is consistent with a low prevalence of ARVD/C, possibly due to under-recognition. Since this is a registry-based study, not all subjects underwent all clinically available tests such as Holter monitoring. It is possible that Holter monitoring was mainly employed in those with mild disease, which may explain a relatively modest PVC count in this cohort. Data on exercise participation were not available. Given recent studies confirming the role of strenuous exercise in phenotypic development of ARVD/C [18, 19], it would be interesting to further investigate exercise participation in elderly ARVD/C subjects. Detailed analysis of the relationship between structural phenotype and age were beyond the scope of this study. Since a large proportion of elderly patients presented with ventricular arrhythmias, only cross-sectional analyses could be performed to determine predictors of arrhythmic events. While we are aware of the broad differential diagnosis of arrhythmias in an elderly population (particularly ischaemic heart disease), we were unable to obtain coronary angiography in all patients. Of note, all patients who did not undergo coronary angiography had no electrocardiographic (exercise stress testing or resting ECG) and CMR evidence of previous myocardial infarction.

## Conclusions

The results of our study show that ARVD/C in elderly patients is characterised by depolarisation abnormalities, frequent ectopy on Holter monitoring, and structural changes on cardiac imaging, while molecular genetic analysis reveals a high percentage of carriers of the phospholamban *p*.Arg14del mutation. Ventricular arrhythmias are typically haemodynamically well-tolerated monomorphic VT and are associated with male sex and proband status. These results suggest that further evaluation and more aggressive risk stratification may be justifiable in male probands surviving beyond 50 years of age. Concomitant CAD remains a source of misinterpretation in the aetiology of monomorphic VT in this elderly age group.
